# Dynamics of contact electrification

**DOI:** 10.1126/sciadv.abg7595

**Published:** 2021-05-26

**Authors:** Mirco Kaponig, Andre Mölleken, Hermann Nienhaus, Rolf Möller

**Affiliations:** Faculty of Physics and Center for Nanointegration Duisburg-Essen CENIDE, University of Duisburg-Essen, Lotharstraße 1, 47057 Duisburg, Germany.

## Abstract

Although the electrical charging of objects brought into contact has been observed for at least 2000 years, the details of the underlying mechanism are still not yet fully understood. The present paper deals with the very basic process of contact electrification between two metals. We have developed an experimental method to follow the charge of a small sphere bouncing on a grounded planar electrode on a time scale down to 1 μs. It reveals that the sphere is discharged in the moment of contact, which lasts about 6 to 8 μs. However, at the very moment of disruption of the electrical contact, it regains charge far beyond the expectation according to the contact potential difference. The excess charge rises with increasing contact area.

## Teaser

A detailed analysis of contact electrification between metals reveals a mechanism leading to unexpectedly high voltages.

## INTRODUCTION

Contact electrification is a ubiquitous phenomenon that occurs whenever two surfaces touch. It is the elementary process of triboelectricity that can be directly observed in daily life. It is responsible, e.g., for lightning in thunderstorms, sandstorms, or volcanic plumes. It is of major concern when handling potentially explosive liquids or dusts. Empirical safety regulations have been established to avoid hazards caused by electric discharges due to triboelectric charging. Although it has been described for more than 2000 years, the underlying mechanisms are still debated.

Basically, three kinds of charge transfer need to be considered (*1*, *2*): transfer of electrons, ions, or material with partial charge. It is generally agreed that, for metal-metal contacts, electrons are transferred between the two surfaces such that the contact potential is established, which is given by the difference of the corresponding work functions. As discussed by Harper (*3*, *4*) and later by Lowell (*5*), the amount of the transferred charge depends on the mutual capacity at the moment when the electric contact is disrupted. The agreement between the prediction based on the work function difference and the observed charge transfer strongly supports the concept of electron transfer for metal-metal contacts.

The situation is less obvious for metal-insulator or insulator-insulator contacts. The role of electron transfer has been elucidated for contacts between a metal and an inorganic insulator (*6*, *7*), substantiated, e.g., by the observation of the temperature-dependent thermionic electron emission. On the other hand, the correlation of weakly bound ions in polymers on the transferred charge provides evidence of ion transfer (*8*). For other combinations of materials, the water from ambient atmosphere leads to a charge transfer that occurs, e.g., for 30% humidity but neither for 0% nor for 100%.

Experiments measuring the local electric field with high resolution reveal that, for polymers commonly used in tribolectricity, the charges may be distributed very inhomogeneously in a mosaic structure of highly charged patches with opposite sign but little macroscopic net effect. Moreover, it could be shown that the formation of these patches is related to a transfer of material (*9*).

Despite the discussion about the detailed mechanism of contact electrification, an empirical tribological series has been established by experiments over the last centuries. Using liquid metal–insulator interfaces, the reproducibility could be greatly improved (*10*, *11*). The yield of contact electrification has been more and more improved, leading to the development of triboelectric nanogenerators (*12*–*14*).

In this work, we present a novel experimental technique that allows us to analyze the process of charge transfer in contact electrification with unprecedented resolution. It could be revealed how the electric potential of a metallic particle bouncing from a metallic surface evolves in time. The temporal resolution allows us to verify the prediction of the generally accepted model for metal-metal contact electrification in that during the mechanic contact, which only lasts a few microseconds, a constant electric potential difference is established.

However, in contrast to the generally accepted concept for metal-metal contacts (*1*, *3*–*5*, *15*), we find that the charge increases with the impact velocity. This has commonly been observed for metal-insulator or insulator-insulator contacts (*16*–*18*), but not for metal-metal contacts. In the experiment, this can lead to unexpectedly high electric potentials for purely metallic contacts, e.g., when a sphere falls from a height of 40 mm and bounces on a plate, an electric potential of up to 10 V is reached.

Moreover, it is revealed that there is no “memory” of the charge before the contact because an electric contact is established during the mechanical contact of a few microseconds. Thereby, the potential of the sphere is reduced to the contact potential of a few tenths of a volt. However, in the very moment when the electric contact breaks, the charge on the sphere establishes a potential of up to 3 V within less than 1 μs. On a much slower time scale, the potential increases further as the distance between the sphere and the plate grows. In the limit of vanishing impact velocity, the charge and the potential diminish to the values predicted by the work of Harper and Lowell (*1*, *3*–*5*).

## RESULTS

**Fig. 1:**
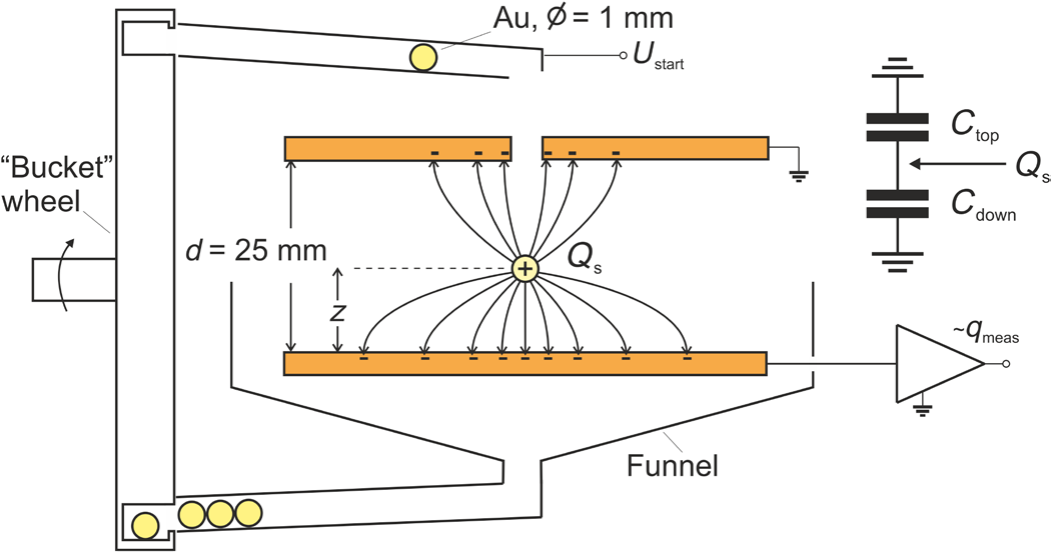
Scheme of the experimental setup. An electric equivalent circuit is displayed in the upper right corner. The charge is “split” between the capacity with the top and the bottom plate.

Matsuyama *et al*. (*19*) studied the charge transfer of particles bouncing on an inclined surface using a contact-free electrostatic detection. We have developed an experimental scheme that not only allows us to measure the charge before and after the contact with the surface but also enables us to follow the dynamics in real time. The setup provides a resolution better than 1 μs in time and about 6000 electrons or 1 fC for the charge (*20*). To study the motion and the contact electrification, gold spheres with 1 mm in diameter are dropped through a small orifice into a parallel plate capacitor. The spheres bounce on the lower plate that is virtually grounded by a charge amplifier measuring the induced as well as transferred charges. A scheme of the experimental setup is shown in Fig. 1. The experiments are performed in vacuum at a base pressure of 2 × 10^−7^mbar.

The signal detected at the lower plate *q*_meas_ has two contributions:

1) The charge on the sphere induces a charge *q*_ind_ of opposite sign in the plate. This charge is taken from the input capacity of the amplifier and the part of the plate, which is not facing the sphere. Hence, it is detected with negative sign.

2) The charge transferred to the sphere upon contact *q*_trans_ is taken from the plate. Hence, it enters with a negative sign as well

$$q_{\text{meas}}=-q_{\text{ind}}-q_{\text{trans}}$$

The induced charges in the upper and the lower plate are split according to the ratio of the capacities *C*_top_ between the sphere and the upper plate and *C*_bottom_ between the sphere and the lower plate (see inset with the equivalent circuit in Fig. 1). For infinite extended plates, the sum of the induced charges is equal to −*Q_s_*, with the charge on the sphere *Q_s_*. Hence

$$q_{\text{ind}}=-RQ_{s},\text{using}\;R=\frac{C_{\text{bottom}}}{C_{\text{top}}+C_{\text{bottom}}}$$

The finite size of the capacitor can be taken into account by including a stray capacitance (see note S1). The charge transferred to the sphere is the difference between the actual charge on the sphere and the initial charge, when it enters the capacitor *q*_trans_= *Q_s_* − *Q*_*s*, initial_ . It follows that

$$q_{\text{meas}}=(R-1)Q_{s}+Q_{s,\text{initial}}=-\frac{C_{\text{top}}}{C_{\text{top}}+C_{\text{bottom}}}Q_{s}+Q_{s,\text{initial}}$$ (1)

As long as the sphere is not too close to one of the plates and neglecting the stray capacity, $R=\frac{C_{\text{bottom}}}{C_{\text{top}}+C_{\text{bottom}}}=\frac{d-z}{d}$, where *d* is the distance between the plates and *z* is the height of the center of the sphere above the surface of the lower plate. This leads to

$$q_{\text{meas}}=-\frac{z}{d}Q_{s}+Q_{s,\text{initial}}$$ (2)

**Fig. 2:**
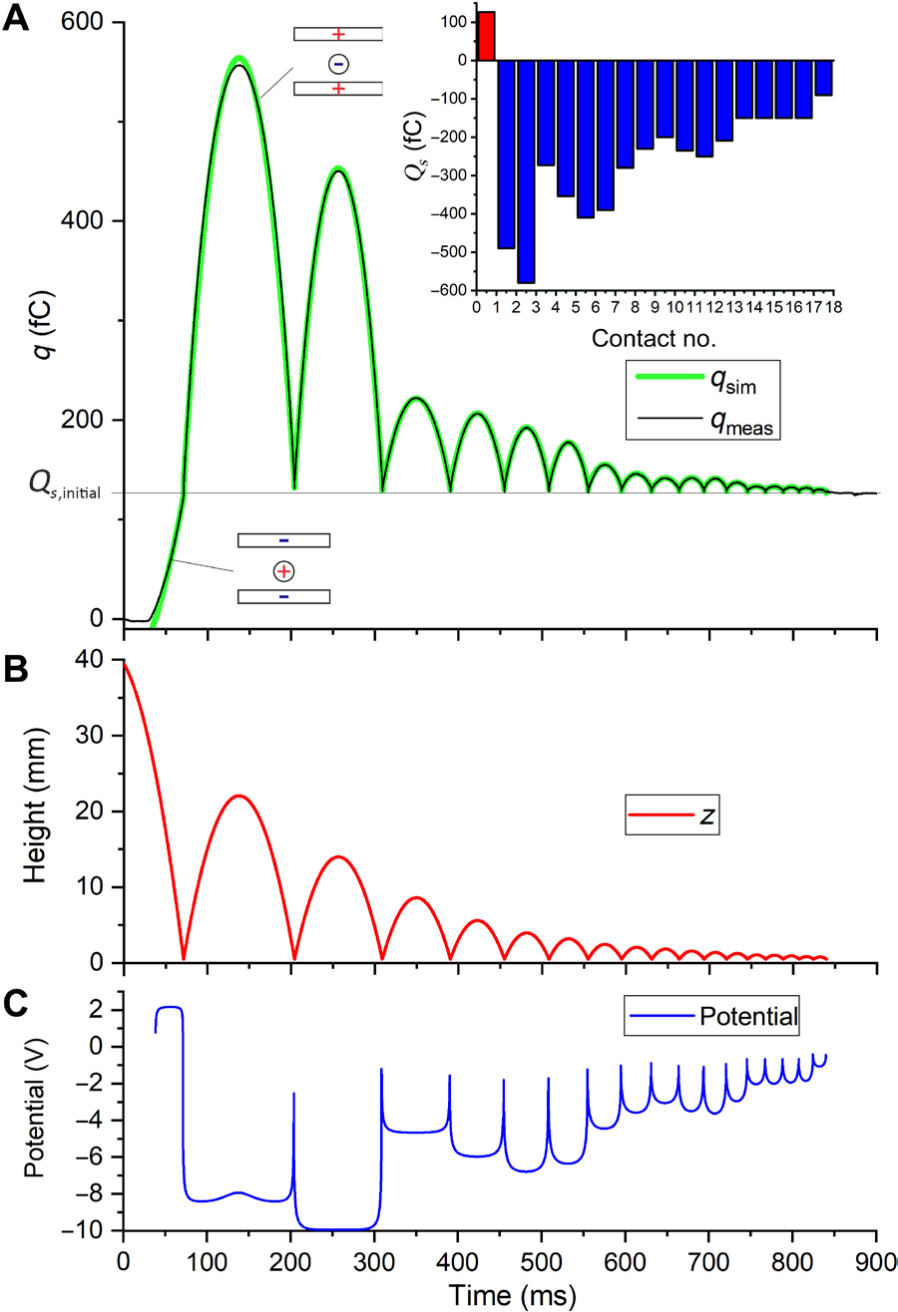
Measurement of the charge on the lower plate of the capacitor and derived quantities. (**A**) The signal measured at the lower plate overlaid to a simulation according to Eqs. 1 and 3. It shows a perfect agreement, except at the very beginning and the top of the first parabola because of the field distortion in the vicinity of the entrance hole, which is not included in the numerical description. On the given scale, the signal noise is barely visible. The histogram in the upper right corner displays the charge on the sphere between the contacts. (**B**) The vertical position of the sphere bouncing on the plate derived from the contact times. (**C**) The potential calculated according to Eq. 4. It reveals that the sphere may reach a voltage of up to 10 V.

**Fig. 3:**
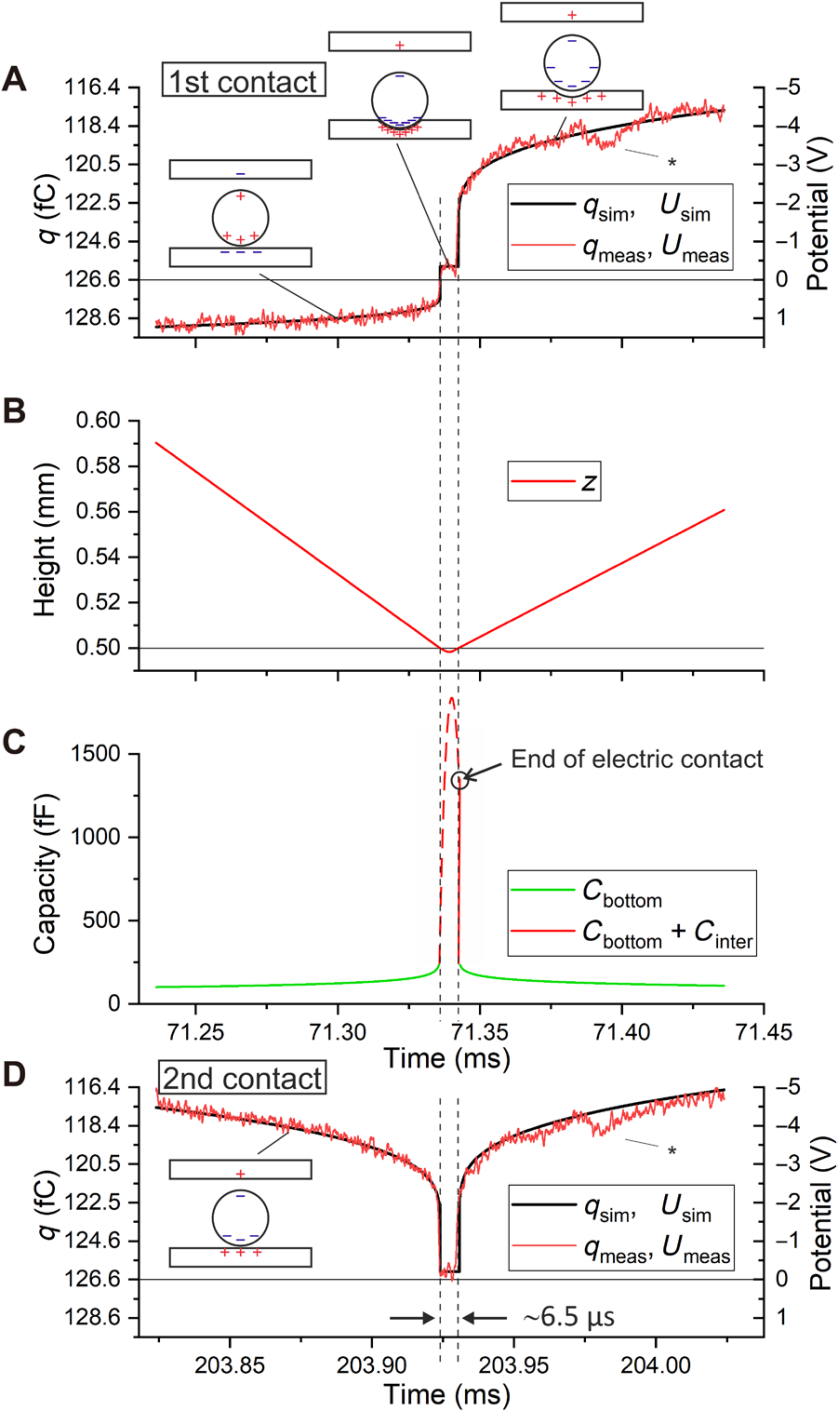
Details of the first and second contact from about 100 μs before and 100 μs after the contact. (**A**) The measured and simulated charge as well as the derived potential for the first contact. The deviation marked by * is due to the “mechanical response” of the plate after the impact of the sphere. The horizontal line corresponds to the initial charge of the sphere or the zero point of the potential. The dashed vertical lines indicate the time interval of the mechanical contact. The plateau of the signal corresponds to the electrical contact. The insets sketch the charge distribution on the sphere and the plates. The relative size of the sphere is strongly exaggerated. The deformation is schematic; in reality, both the sphere and the surface are deformed. (**B**) The corresponding height of the sphere. The motion before and after the contact is almost linear on the short time scale. (**C**) The calculated capacity before and after the contact by the green line. During the contact, a tentative value proportional to the contact area is sketched by the dashed red line. The arrow points to the value of the capacity at the very moment when the electric contact is broken. It is assumed that the capacity is enhanced relative to the ideal geometry because of the deformation of the contact area by creating relatively large adjacent surfaces. (**D**) The measured and calculated charge as well as the derived potential for the second contact.

Figure 2 displays the signal for a gold sphere bouncing more than 15 times on the lower plate of the capacitor that is made of copper. The trajectory of the sphere consists of segments of free fall starting and ending at a contact with the plate (Coulomb forces may be neglected; see note S2). A close inspection of the signal allows the identification of the moments of contact by abrupt changes of the measured charge *q*_meas_ (see, e.g., Fig. 3).

The time ∆*t* between two contacts completely determines the segment of the trajectory (see note S3). Figure 2B displays the height as a function of time.

Hence, using Eq. 2, the charge on the sphere can be uniquely evaluated from *q*_meas_ for each segment of the trajectory. The corresponding values are displayed in the inset of Fig. 2A.

By applying a voltage *U*_start_ at the ramp guiding the sphere to the entrance of the capacitor, the sphere is positively charged before it enters the capacitor. During the first contact, it becomes negatively charged. In the subsequent contacts, the charge changes, but it always remains negative. The magnitude of the charge is unexpectedly high, but there is an overall decreasing trend for the contacts to follow (see note S4 and fig. S1).

The experiment has been repeated with different initial charges, positive, negative, and no charge at all. In all cases, the sphere becomes negatively charged in the first and the following contacts, irrespectively of the initial charge. Apparently, there is no memory for the charge before the contact.

For a detailed quantitative analysis, an additional contribution to the capacities has to be included when the sphere comes close to the plate, because the charge on the surface of the conducting sphere will redistribute owing to the Coulomb attraction. Hence, the capacity to the plate nearby increases. It has been shown that this can be corrected in good approximation by an additional term (*21*)

$$C_{\text{bottom}}=(\frac{d-z}{d}+0.5∙\text{ln}(1+\frac{r}{a}))C_{0}$$ (3)

where *C*_0_ = 4πε_0_*r* is the capacity of a conducting sphere in free space, with $\varepsilon_{0}=8.854\times 10^{-12}\frac{F}{m}$ being the permittivity of the vacuum, *r* being the radius of the sphere, and *a* = max(*z* − *r*, *d*_min_). The latter expression ensures a finite positive value of $\frac{r}{a}$. As discussed in (*3*–*5*, *15*), the effective separation *d*_min_ depends on the roughness of the surfaces, which prevents the sphere from coming as close to the plate as in the ideal geometry. On the basis of the ln function in Eq. 3, this value is not critical. We find a good agreement for *d*_min_ = 100 nm. This has been precisely verified experimentally (*22*).

By replacing *z* by *d* − *z* , *C*_top_ is obtained.

Using these capacities, a very accurate description of *q*_meas_ is obtained, as can be seen in Fig. 2A and, in particular, in Fig. 3 (A and D).

A key to the understanding of the contact electrification is the potential of the sphere, which is given by

$$U=\frac{Q_{s}}{C_{\text{top}}+C_{\text{bottom}}}$$ (4)

It is readily calculated using Eq. 3 for *C*_top_ and *C*_bottom_ and displayed in Fig. 2C. If the sphere is more than one radius apart from the plate, the sum of the capacities is close to *C*_0_ and the potential is almost constant for each segment of the trajectory. Corresponding to the high magnitude of the charge on the sphere, a potential of several volts is found, which is unexpectedly high for a purely metallic system.

However, to evaluate the potential for the very moment of the contact, Eq. 4 fails, because *C*_bottom_ can only be guessed. Using Eq. 1, the unknown *C*_bottom_ can be eliminated and we obtain

$$U=\frac{Q_{s,\text{initial}}-q_{\text{meas}}}{C_{\text{top}}}$$ (5)

Because *C*_top_ can be calculated precisely, this allows us to evaluate the potential based on the measured charge *q*_meas_. A descriptive interpretation of Eq. 5 is that the numerator is the negative-induced charge in the top plate. Dividing by the corresponding capacity provides the potential.

Figure 3 displays two sections of 200 μs during the first and the second contact to the plate. Figure 3 (A and D) displays the potential according to Eq. 5; the scale on the left side for the measured and calculated charge is adopted assuming a constant value of *C*_top_, which is correct to within 2%. The curvature of the signal before and after the contact is well described by the logarithmic increase of the capacity according to Eq. 3. The deviation at about 40 to 60 μs after the contact is most likely due to a mechanical “response” of the plate following the impact of the sphere. It decreases with decreasing impact velocity (see note S6 and fig. S5).

The dashed lines indicate the time of the mechanical and electrical contact, which lasts about 6.5 μs. For comparison, the indentation of the sphere is simulated by Hertzian contact mechanics using the elastic constants for copper and gold, respectively (see note S5 for details). The calculated contact time agrees to the electrical contact time. Despite the small mass of the sphere of 10 mg, the peak force exceeds 4 N for the first contact. Scaled according to the radius of the spheres, our data also agree with data observed using a wire attached to the sphere to drive an electric current during contact (*23*, *24*) or by optical techniques (*25*).

Because Fig. 3 shows that the electric potential is almost constant during the contact, it is safe to assume that a conducting electric contact is established. There is a variation between the different contacts, but the potential is always negative by a few tenths of a volt, which is expected for the contact potential between gold and copper (work function: gold, 5.1 to 5.4 eV; copper, 4.5 to 4.9 eV). The data for all contacts are displayed in fig. S1.

Using Eq. 3, *C*_bottom_ = 250 fF may be calculated for the smallest distance, which has been set to 100 nm taking the roughness of the surfaces into account in accordance with the work by Lowell (*5*). Even assuming a rather large contact potential of −0.4 V, this would lead to a charge of 100 fC only. However, that is not sufficient to explain the charge after a contact observed in our experiment. Moreover, there is no reason why it should depend on the impact velocity.

Before the measurement, the experiments have been performed at ambient conditions, leading to a layer of water and other contaminants depending on the humidity in the room. The data are somewhat less consistent because of spurious leakage currents. However, the findings essentially agree to the presented data obtained at a pressure of 2 × 10^−7^mbar. At the latter, the adsorbate layer should be reduced to a few monolayers. Hence, we conclude that the excess charge induced by contact electrification is not due to effects of adsorbates.

## DISCUSSION

To explain our observations, we propose the following model: During the contact, the contact area raises, e.g., to 0.00027 mm^2^ for the first contact. At the interface, an enormous capacity will be formed because of the minimal distance between the charges (as sketched in the inset of Fig. 3). A hypothetical interface capacity is drawn in Fig. 3C. This capacity will be charged to the contact potential. These charges bound to the interfaces are most likely in the order of picocoulombs or more. When the contact breaks, the two adjacent surfaces of the plate and the sphere caused by the plastic deformation will fit almost snugly, leading to a larger area at close separation and a larger capacity than in the ideal geometry. This is indicated by the sketch in the upper right part of Fig. 3A and by the circle in Fig. 3C. It is to be expected that the size of this area depends on the velocity of the sphere. This is corroborated by the decreasing trend of the charges observed for the charges after subsequent touches of the sphere, shown in the inset in the upper right corner of Fig. 2. In the Supplementary Materials, a large dataset is discussed (fig. S1), revealing a possibly linear correlation between the transferred charge and the contact area including an impact independent offset of 100 to 200 fC. The latter indicates that, for vanishing impact velocity, our results converge to the prediction by the model of Harper and Lowell (*3*–*5*).

Moreover, for comparison, a measurement using identical materials, i.e., a gold sphere and a gold plate, has been performed (see note S7 and fig. S6). As expected, only minimal charges due to the contact electrification are found.

In summary, the charge and the potential of a sphere bouncing on a plate could be studied in great detail with sub-microsecond time resolution. It reveals that the charge transfer is limited to the short mechanical contact with a duration of a few microseconds. There is no accumulation of charge in subsequent contacts because the potential is reset to the contact potential at each contact.

We have found an impact-dependent mechanism that increases the charge transferred by a metal-metal contact beyond the model by Harper and Lowell. For a metallic sphere bouncing from a metal plate, the potential may reach up to 10 V. We propose that this is due to a deformation of the contact area that goes along with an increase of the capacity between the sphere and the plate at the moment when the electrical contact is disrupted. This will be important in contact electrification and triboelectricity involving insulators as well (*26*), because an enlargement of the contact area by deformation will lead to an enhanced charge transfer in a similar way. However, for the latter, the charge will not be “reset” in subsequent contacts because of the lacking electric conductivity and it may accumulate with each contact (*17*).

## MATERIALS AND METHODS

The spheres used in the experiment are made by granulation from 995 gold (99.5% gold). Immediately before placing the spheres in the vacuum chamber, they were ground by a self-built ball rolling machine to improve the shape and eventually further clean the surfaces. Figure S7 shows images taken by scanning electron microscopy of gold spheres before (fig. S7A) and after (fig. S7B) grinding. It clearly shows the improvement of the shape. The circles indicate roughly the size of the maximal contact (see note S5 for the calculation by Hertzian model) for the first and one of the later (10th) contacts.

The plates of the capacitor are made of copper, polished and cleaned by ultrasonication. For each measurement, a single sphere is picked by the “bucket” wheel and lifted to a “canal,” which ends with two 90° turns above the circular opening in the upper plate of the capacitor. The turns almost stop the motion of the sphere, such that it drops with a negligible horizontal component of the velocity. Before entering the capacitor, it crosses a light barrier that triggers the measurement.

An essential part is the specially developed charge amplifier that allows measuring the voltage or charge at the input from DC to about 2 MHz with a gain of 12. A patent is filed for the electronic scheme (German Patent and Trade Mark Office No. 10 2019 111 694.5). For the given experiment, it is split into an input stage mounted within the vacuum chamber close to the capacitor plate and external part with lower impedance. Connected to the capacitor, the input capacity amounts to 22 pF, yielding a resolution about 1 fC. After warmup, there is an input offset current in the order of 10^−14^A . To avoid large offsets, the input is reset shortly before each measurement. The data are acquired using a Picoscope 5444D digital scope using a sampling rate of 31.25 MHz.

## SUPPLEMENTARY MATERIALS

Supplementary material for this article is available at http://advances.sciencemag.org/cgi/content/full/7/22/eabg7595/DC1
